# Religion and survival among European older adults

**DOI:** 10.1007/s10433-023-00789-4

**Published:** 2023-10-30

**Authors:** Konstantinos Christopoulos

**Affiliations:** University of Piraeus, 80 Karaoli & Dimitriou Street, 185 34 Piraeus, Greece

**Keywords:** Longevity, Mortality, Religiousness, SHARE, Survival analysis

## Abstract

There are several pathways through which religion can affect longevity. Previous research, predominately from North America, has shown decreased mortality risk for participants that attended religious services. This study aims to examine the association between religion and all-cause mortality in a large sample of older European adults, comparing religious affiliations, and using prayer frequency as well as frequency of participation in a religious organisation as measures of religiousness. To this end, a total of 16,062 participants from the Survey of Health Ageing and Retirement in Europe were employed for a survival analysis (median follow-up 11.3 years; 3790 recorded deaths). Following a religion was negatively associated with mortality regardless of demographic and socioeconomic factors (HR = 0.81; 95% CI 0.74–0.89). Large differences in the median survival of participants from different religious affiliations can be mostly attributed to demographic and socioeconomic factors. Both frequency of prayer and religious participation exhibited a significant positive dose–response relationship with survival despite adjustments, although the results for religious participation were more profound. Changes on the religiosity levels of the European population will require additional research on the subject in the future.

## Introduction

A connection between religion and health exists since ancient times. Although medicine has come a long way from the healing snakes of Asclepius—god of medicine—and society has probably lost most of her faith in faith healing, there is strong evidence that religion has a positive effect on human health. This effect is often mediated by societal, psychological, and behavioural factors, all proven to affect the health and longevity of the population (Morton et al. 2017). In theory, the more religious the person, the more he or she adheres to the religious traditions and dogma. This fact leads to several pathways through which religion (or religiosity) can influence the health of older individuals.

Social relationships have been associated with increased survival in older adults (Rodriguez-Laso et al. 2007). The societal path, through which religious individuals may interact with others, during church attendance for example, or receive support (financial or emotional) from members of their church, is one potential mechanism. There is also the psychological path, through which individuals may experience reduced anxiety and/or depression with prayer and belief, although this view was challenged in a recent study, at least for older Europeans (Van Herreweghe and Van Lancker 2019). Perhaps the most important is the lifestyle path. An example is when a religion prohibits the consumption of certain foods, beverages, or substances, like alcohol, leading to lower likelihoods of consumption (Adnum et al. 2022). Of course, these are no strict boundaries and the paths indeed intertwine.^1^

The plethora of published studies and books over the past decades indicate that the subject has been largely studied from multiple scientific points of view (e.g. psychology sociology, gerontology, epidemiology, and other). The most recent review to date on the subject, including the highest-quality studies, recommends that more attention be paid to spirituality in healthcare and health research (Balboni et al. 2022).

Nevertheless, mental and physical health outcomes have been studied extensively. Regarding mental health, studies have focused on depression (Braam and Koenig 2019; Van Herreweghe and Van Lancker 2019), suicide (VanderWeele et al. 2016), anxiety (Shreve-Neiger and Edelstein 2004), psychosis (Menezes and Moreira-Almeida 2010), and substance abuse (Grim and Grim 2019). Koenig (2009) offers a review on religion and mental health outcomes. In a meta-analysis of longitudinal studies on mental health, Garssen et al. (2021) found a positive significant, albeit small, association with religious activity participation and religious importance. The protective associations concern mainly depression and suicide (Balboni et al. 2022). Multiple studies show positive associations with physical health as well (Gonçalves et al. 2017; Yeager et al. 2006), but results are often mixed (Balboni et al. 2022).

Caution is necessary when evidence comes from cross-sectional studies which happen to have the unfortunate combination of volunteer bias and self-reported outcomes. Studies also show that the potential effect of religion on health is highly variable between countries, races, and genders, and it may be moderated by local societal and cultural norms (Stavrova 2015; Zimmer et al. 2019). More recently, religion was studied within the context of the COVID-19 pandemic. Themes included the potential health risks and benefits (Knight et al. 2021), the spiritual care needs of COVID-19 patients (Şahan and Yıldız 2022), the mortality of different religious affiliations (Gaughan et al. 2021), as well as the effect online religious participation on health (Shiba et al. 2023).

Despite inherent difficulties in exposure measurement, longitudinal studies on mortality provide some of the most robust evidence. Chida et al. (2009) offer a systematic review and meta-analysis regarding all-cause mortality. They find reduced mortality risk for religious followers in healthy samples but not when examining population samples with diseases. They also make a case for publication bias. Balboni et al. (2022) offer the latest review on religion/spirituality and health to date using a multidisciplinary Delphi panel approach. All-cause mortality was reviewed excluding retrospective and cross-sectional studies and strongly supports the idea that service attendance is associated with a lower risk of mortality, and probably this is a dose–response association.

The majority of the research concerns participants from US locations (Bagiella et al. 2005; Bruce et al. 2022; Gillum et al. 2008; Helm et al. 2000; Hill et al. 2005; Idler et al. 2017; Hummer et al. 1999; Kim et al. 2015; Li et al. 2016; McCullough et al. 2009; Musick et al. 2004; Schnall et al. 2010; Sullivan 2010; VanderWeele et al. 2017), but studies from other countries such as Denmark (Ahrenfeldt et al. 2023; La Cour et al. 2006), Ireland (O’Reilly and Rosato 2008), Finland (Teinonen et al. 2005), and China (Zhang 2008) also demonstrate a negative association between church attendance and all-cause mortality. This finding remains consistent when examining older populations (Ahrenfeldt et al. 2023; Bagiella et al. 2005; Bruce et al. 2022; Helm et al. 2000; Hill et al. 2005; Teinonen et al. 2005; Idler et al. 2017; Zhang 2008).

For other measures of religion or religiosity, such as prayer frequency, religious affiliation, and religious salience, the results are mixed. Studies often find null (Idler et al. 2017; Kim et al. 2015; VanderWeele et al. 2017) or smaller and less significant associations compared to service attendance (Schnall et al. 2010). Idler et al. (2017) using data from the Health and Retirement Study (HRS) found that those who regard religion to be very important had a small *increase* in the risk of death.

National or regional studies have very limited religious affiliations, and sometimes only denominations of the same religion. But focusing on diverse affiliations is important in discovering disparities and examining their causes, since studies have shown differences in mortality (Kim et al. 2015; O’Reilly and Rosato 2008; Sullivan 2010). Another issue is the measurement of religiosity. Church attendance and prayer frequency are the most common approaches, but depending on the heterogeneity of the sample, both instruments can have serious limitations.^2^ As mentioned before, there is plenty of evidence from US populations with church attendance as the instrument of choice. On the other hand, European populations with diverse affiliations and prayer frequency as an instrument have been understudied.

This study revisits the subject of religion and longevity in the context of an older European population pooling a total of 16,062 individuals from 11 countries. Three hypotheses are tested: (1) whether having any religious affiliation or partaking in a religious organisation influences survival, (2) whether the religious affiliations under study are associated with survival, compared to not following a religion, and (3) whether the amount of faith or ‘religiousness’, measured by the frequency of prayer as well as the frequency of partaking in a religious organisation, is related to survival. The last hypothesis aims to test whether there is a dose–response, which would corroborate potential findings. Data from the Survey of Health and Ageing and Retirement in Europe (SHARE) were extracted and analysed to this end.

## Material and methods

### Survey and study sample

The SHARE project is a longitudinal survey that samples older European adults from several countries periodically. It uses computer-assisted personal interviews (CAPI) to collect the data. The instrument has been validated and translated by pre-testing on a probability subsample. Participation rates are highly variable between countries but remain very high compared to similar surveys (see Bergmann et al. 2017, for details). It has undergone review from a University’s ethics committee, and all participants have given their written consent. For more information on SHARE, see Börsch-Supan et al. (2013).

**Fig. 1:**
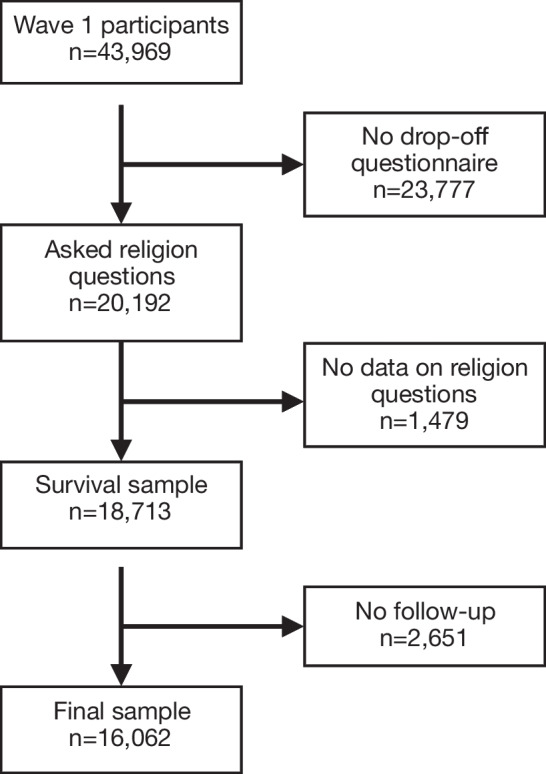
Flowchart for the selection of study participants

The study sample consists of 16,062 participants from 11 countries from the first wave of the SHARE project (approximately from 2004)—the only wave where all these questions regarding religion were asked. Although not a traditional cohort study, SHARE can be regarded as a follow-up survey. The 11 countries represented in this study are described in Table 1. Exclusion criteria include: values missing for all religion questions/variables examined, and no follow-up time. Figure 1 provides a flowchart of the sample selection. Data on the birth, survey entry, and death time were retrieved from the survey modules.^3^

**Table 1:** Study sample countries of origin

Country	*N*	%
Austria	1219	7.59
Germany	1258	7.83
Sweden	1908	11.88
Netherlands	1616	10.06
Spain	1356	8.44
Italy	1246	7.76
Denmark	1138	7.09
Greece	1947	12.12
Switzerland	616	3.84
Belgium	2283	14.21
Israel	1475	9.18

### Exposure variables

The five variables examined in total come from four questions. Regarding religious affiliation, the question was the following: ‘What religion do you belong or feel attached to mostly?’ [Protestant (e.g. Lutheran or Anglican church); Protestant (evangelist) free church/other protestant; Roman Catholic; Greek or Russian Orthodox; Jewish; Islam; Hinduist; Buddhist; Esoteric, New Age; Other (Please specify):...; I do not belong or feel attached to any religion]. Answers were merged by SHARE into seven categories [Protestant; Catholic; Orthodox; Jewish; Muslim; Other; None] which were used in the analysis. From this question, an indicator variable was created, where zero those who chose ‘None’, and one otherwise.

Regarding religiosity, participation in a religious organisation was measured by the following two questions: ‘Have you done any of these activities in the last month?’ with ‘Taken part in a religious organization (church, synagogue, mosque, etc.)’ [Yes; No] as the relevant answer, and the relative frequency of that participation by the question ‘How often in the last four weeks have you taken part?’ [Almost daily; Almost every week; Less often]. For those that selected ‘No’ in the former question, a new category ‘No participation’ was created in the variable that was created from the latter question. Finally, the question ‘Thinking about the present, about how often do you pray?’ [More than once a day; Once daily or almost daily; A couple of times a week; Once a week; Less than once a week; Never] was used additionally as a proxy for religiousness. Religious affiliation and prayer frequency questions come from a drop-off self-completion questionnaire especially designed for sensitive subjects with very high participation rates (about 80% on average).

### Covariates

Demographic covariates such as age at baseline interview, gender, marital status, and country of residence were used in the analysis. Socioeconomic variables like education, household financial management (henceforth, household finances), and receiving social support from outside the household were also employed as covariates.

To control for the physical and mental health, the number of: chronic conditions, activities of daily living (ADL), and instrumental activities of daily living (IADL) were included, as well as the Euro-D depression scale (Prince et al. 1999). Additionally, the Body Mass Index (BMI) as well as three behavioural risks factors, smoking, drinking, and physical inactivity were also used as controls. All covariates (including exposures) are self-reported and measured at baseline (in 2004–2005). For details on the available responses, readers can refer to Table 2.

### Identification strategy

**Fig. 2:**
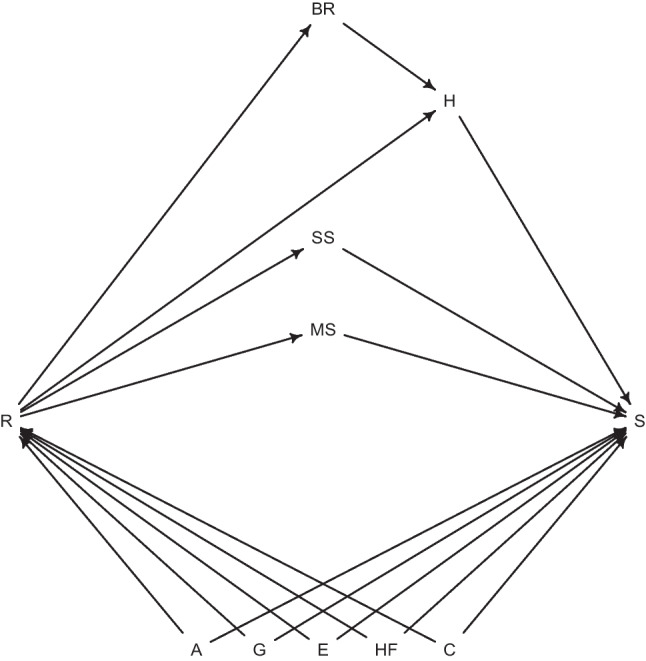
Directed Acyclic Graph for the study of religion and survival. *Notes* R = religion, S = survival, A = age, G = gender, HF = household finances, E = education, C = country, BR = behavioural risks, H = health, MS = marital status, and SS = social support. The graph was created using the *R* package ‘*dagitty*’

Covariates were chosen in an effort to reduce confounding to a minimum and explore potential mediating paths with the help of Directed Acyclic Graphs (DAGs). One such graph is presented in Fig. 2. The graph represents the assumptions made for this analysis. For the sake of readability, arrows between variables other than the exposure (R) and outcome (S) were omitted and some covariates were grouped. Moreover, the DAG assumes no unobserved confounding and no measurement errors.

The DAG additionally assumes that religion only influences survival indirectly through the four aforementioned paths that are represented by BR (behavioural risks)—which in turn influence health (H)—SS (social support), H (health directly, via mental health mainly), and marital status (MS). It also regards, age (A), gender (G), household finances (HF), education (E), and country (C) as confounders.

Demographic, socioeconomic, and health related variables are expected to have a different distribution^4^ between religious and non-religious individuals (Hayward et al. 2016; Litwin et al. 2017; Mueller and Johnson 1975), and their role in mortality is well established. Health was not considered as a confounder in this DAG since the distributional differences in health variables between the two groups in the sample were trivial (more on the "Results" section) but is considered in the analysis of the religiosity variables since previous studies have detected health selectivity in church attendance (Hummer et al. 1999). These assumptions of course do not rule out the scenario that some of the mediators may also be confounders.

### Statistical analysis

A survival analysis was performed in order to test the three hypotheses. This method is used to model time-to-event data. It is very common in fields like epidemiology, but has applications in many other fields (where it goes by different names) such as sociology (e.g. time to commit a crime after being released from prison), labour economics (e.g. time to find a new job), operations research (e.g. time until a machine breaks down), and other. For more details on the method, see Kalbfleisch and Prentice (2011).

The time scale used was the attained age, since it accounts for the different time participants entered the study (left-truncation) and is more appropriate for the survival analysis of older adults (Lamarca et al. 1998). Gompertz proportional hazards (PH) models were employed to obtain hazard ratios (HR) and 95% compatibility intervals (CI). The HR shows the proportionate change in the hazard given a change in a covariate. The proportionality assumption was tested using the Schoenfeld residuals separately for the five religion variables used. A constant hazard was assumed over time. Cluster robust standard errors were used at the household level to account for the non-independence.

Missing values (MV) were imputed using SHARE’s imputations for variables where these were available (all except the exposures and the social support variable). Mean imputation was performed for the remaining small gaps ($n<7$). For the social support variable, a household approach—where persons in the same household received the same value if the value was missing—was taken. Nevertheless, $n=264$ MV remained and were dealt with pairwise deletion as was the case with the MV for the exposures: religion indicator and affiliation ($n=89$), prayer frequency ($n=167$), and partaking in religious organisation ($n=41$). In the case of missing values for the time of death, individuals were censored at the time last interviewed.

The survival analysis is divided in religious affiliation and religiosity exposures. Four models were built to test the hypotheses. Model 1 adjusts for age and gender; Model 2 additionally adjusts for education and household finances (and health variables for the religiosity exposures; Table 5); Model 3 adjusts also for country to provide the no pooling or within-country estimate. Model 4 adjusts for variables in Model 3 plus the variables that are hypothesised to be mediating the effect of religion, namely, marital status, social support, behavioural risks, and health variables (only for the analysis of religious affiliation; Table 4). BMI, ADL, and IADL were modelled with a quadratic term. Stratified analyses by gender were also performed for the two indicator exposure variables.

## Results

### Sample and survival descriptives

Unsurprisingly, the vast majority of the sample consists of Christians (76.58%). Catholics were the most prevalent group (40.06%), while Muslims the least (1.28%). Protestants (24.33%), Orthodox (12.18%), Jewish (7.25%), Other religions (2.13%), and without religious affiliation (12.77%) make up the rest of the sample. Conversely, only 14% reported participation in a religious organisation in the last month.

Table 2 displays a selective comparison between those with and without religious affiliation based on the effect size (ES) of standardised differences, where lower values signify higher distributional overlap (Cohen 1988). The two groups are almost identical in BMI, ADL, and social support (ES $\approx$ 0). Small differences (ES < 0.2) exist in the depression score (Euro-D), number of chronic conditions, IADL, drinking, and physical inactivity. Medium differences (0.2 < ES < 0.5) exist in marital status, age, gender, household finances, smoking habits, and education.

In simpler terms, participants with religious affiliation were older on average, consisted of more females than males, and had fewer years of education. In terms of household finances, non-religious people reported to get by more easily. Regarding health risk factors, participants with religious affiliation drink and smoke less, but have more physical inactivity. Medical variables show that participants with religious affiliation have higher scores on the Euro-D scale (meaning more depression), while the number of chronic conditions is about the same.

**Table 2:** Selected demographic, socioeconomic, behavioural, and medical characteristics between participants with and without religious affiliation

		With	Without	Std.diff.
		*N* = 13,934 (87.23%)	*N* = 2039 (12.77%)	
Demographic				
Age (at baseline)	Mean (SD)	64.03 (10.27)	61.41 (9.31)	0.267
Gender	Male	43%	54.4%	0.228
	Female	57%	45.6%	
Socioeconomic				
Education (in years)$\dagger$	Mean (SD)	9.86 (4.31)	11.88 (3.81)	0.498
Financial management of household	With great difficulty	11.3%	6.7%	0.307
	With some difficulty	28.1%	19.5%	
	Fairly easily	33.3%	35.7%	
	Easily	27.3%	38.1%	
Behavioural risks				
Smoking	Currently smoke	18.8%	25.6%	0.290
	Never smoked daily for at least one year	53.6%	39.4%	
	Have stopped	27.6%	34.6%	
Alcohol consumption	2 glasses 5–6 days a week or everyday	10.7%	17%	0.183
Physical activity	Never vigorous nor moderate	9.4 %	6.1%	0.122
Medical				
BMI ($\textrm{kg}/\textrm{m}^2$)	Mean (SD)	26.50 (4.27)	26.34 (4.37)	0.039
Depression (Euro-D)$\ddag$	Median (IQR)	2 (3-1)	1 (3-0)	0.099
Chronic conditions (count)	Median (IQR)	1 (2-0)	1 (2-0)	0.119

*SD* standard deviation, *IQR* inter quartile range, *Std.diff.* standardised difference, calculated using the *R* package ‘*stddiff*’

$\dagger$ Years of education derived from ISCED-97. $\ddag$ Euro-D values range from 0 to 12, with higher values signifying worse mental heath

During 163,951 years of follow-up, 3790 deaths were recorded and the mortality rate was 23.16 deaths per 1000 person-years. The median follow-up was 11.33 years, and median survival was 86.08 years. Orthodox Christians had the longest median survival (87.08), while Muslims the shortest (80.33). Nevertheless, these values concern unadjusted estimates that can be confounded by a variety of factors. For more details on religion specific survival, see Table 3.

**Table 3:** Unadjusted median survival with 95% compatibility intervals and mortality rates for different religions

	Median survival (in years)	Mortality rate (per 1000 person-years)
Full sample	86.08 (85.75–86.50)	23.16
Any religion	86.17 (85.83–86.58)	23.74
No religion	85.67 (84.42–87.17)	18.73
Protestant	85.92 (85.17–86.67)	25.03
Catholic	86.33 (85.83–87.00)	23.07
Orthodox	87.08 (86.17–87.58)	22.82
Jewish	85.00 (82.75–86.67)	26.13
Muslim	80.33 (78.17–82.00)	25.97
Other	84.83 (82.83–88.83)	18.59

### Religious affiliation

Comparing those that reported a religious affiliation versus those who did not, a statistically significant reduction in risk was observed after adjustment for the hypothesised confounders in both the pooled (Model 2) (HR = 0.89; 95% CI 0.80–0.99) and within-country model (Model 3) (HR = 0.90; 95% CI 0.81–1.01). Adjusting for the hypothesised mediators (Model 4) completely attenuated the effect. In the stratified analysis (results available on request), males had qualitative similar significant HRs but slightly lower coefficient values. For female, HRs were higher and insignificant.

Regarding specific religious affiliations compared with those without one, a statistically significant protective association was found for Protestants (HR = 0.88; 95% CI 0.77–0.99) but only in the within-country model (Model 3). On the contrary, for Catholics, a significant protective association was found only before adjusting for country (Model 2) (HR = 0.85; 95% CI 0.75–0.95). This was also the case for Orthodox Christians (HR = 0.80; 95% CI 0.70–0.92). On the other hand, Muslims had a significant increased risk in the basic (Model 1) and pooled (Model 2) model (HR = 1.39; 95% CI 0.97–1.99) but the association became insignificant with the addition of country fixed-effects (Model 3) and in the ‘direct effect’ model (Model 4). No significant association was found for Jewish or Other affiliations or in the model which included the hypothesised mediators in general (Model 4).

Since Christian Orthodox and Jewish participants come predominately from Israel and Greece, respectively, a sensitivity analysis was performed as per the Editor’s instructions. Participants from the aforementioned countries were dropped as were those who were not Protestants or Catholics. The analysis consisted of 12,205 observations and 2809 events. As can be seen in Table 4, the results are almost identical.

**Table 4:** Gompertz PH all-cause mortality hazard ratios and 95% compatibility intervals for religious affiliation ($N=19,973$; Events = 3794)

	Model 1		Model 2		Model 3		Model 4	
	HR	CI	HR	CI	HR	CI	HR	CI
No religion (ref.)								
Any religion	0.95	(0.86–1.05)	0.89**	(0.80–0.99)	0.90*	(0.81–1.01)	0.99	(0.89–1.11)
No religion (ref.)								
Protestant	0.95	(0.84–1.06)	0.96	(0.85–1.08)	0.88**	(0.77–0.99)	0.98	(0.86–1.12)
Catholic	0.93	(0.83–1.04)	0.85**	(0.75–0.95)	0.92	(0.81–1.05)	1.01	(0.89–1.16)
Orthodox	0.95	(0.84–1.07)	0.80***	(0.70–0.92)	1.31	(0.74–2.31)	1.32	(0.78–2.23)
Jewish	0.99	(0.86–1.15)	0.94	(0.81–1.09)	0.87	(0.62–1.21)	0.78	(0.55–1.12)
Muslim	1.63***	(1.12–2.37)	1.39*	(0.97–1.99)	1.27	(0.81–2.02)	1.16	(0.74–1.81)
Other	0.97	(0.74–1.26)	0.86	(0.66–1.14)	0.87	(0.64–1.18)	0.89	(0.63–1.26)
No religion (ref.)								
Protestant	0.96	(0.86–1.08)	0.97	(0.86–1.09)	0.89*	(0.78–1.01)	1.00	(0.87–1.15)
Catholic	0.95	(0.85–1.06)	0.86**	(0.77–0.97)	0.94	(0.83–1.07)	1.05	(0.92–1.21)

Model 4 has $N=15,711$ and Events = 3709 due to missing values. The sensitivity analysis has $N=12,205$ and Events = 2809 for Models 1–3 and $N=12,008$ and Events = 2767 for Model 4

*, **, *** Denote the significance level at 10%, 5% and 1%, respectively. Model 1 adjusts for age and gender. Model 2 adjusts for factors in Model 1 + education and household finances. Model 3 adjusts for factors in Model 2 + country of residence. Model 4 adjusts for factors in Model 3 + potential mediators, namely, depression, No. chronic conditions, BMI, marital status, social support, smoking, physical inactivity, ADL, IADL, and alcohol consumption

### Religiosity

Partaking in a religious organisation was significantly associated with decreased mortality risk in all specifications, even in the one that aimed at blocking the indirect paths (Model 4) (HR = 0.86; 95% CI 0.78–0.95). In the stratified analysis (results available on request), both genders had qualitatively similar significant HRs in all specifications, although males had again lower HRs than females.

Monthly participation frequency showed quantitatively similar statistically significant associations in all specifications. It also exhibited a dose–response with HRs lowering as the frequency of participation increased. Lower statistical significance for those who participated almost daily is probably the result of their small sample size ($n=220$). On the other hand, frequency of prayer only showed statistically significant prophylactic associations of lesser magnitude for those that pray at least once per day or almost daily. The results appear in Table 5.

**Table 5:** Gompertz PH all-cause mortality hazard ratios and 95% compatibility intervals for religiosity exposures (participation: $N=16,021$, Events = 3769; prayer: $N=15,886$, Events = 3733)

	Model 1		Model 2		Model 3		Model 4	
	HR	CI	HR	CI	HR	CI	HR	CI
*No participation (ref.)*								
Participation	0.78***	(0.73–0.85)	0.81***	(0.74–0.89)	0.80***	(0.73–0.88)	0.86***	(0.78–0.95)
*No Participation (ref.)*								
Less often	0.84**	(0.72–0.97)	0.85**	(0.73–0.99)	0.84**	(0.72–0.98)	0.88*	(0.75–1.03)
Almost every week	0.76***	(0.68–0.86)	0.80***	(0.71–0.90)	0.79***	(0.70–0.89)	0.86**	(0.76–0.98)
Almost daily	0.72**	(0.56–0.93)	0.77**	(0.61–0.99)	0.79*	(0.61–1.01)	0.80*	(0.63–1.03)
*Never pray (ref.)*								
<1/week	0.94	(0.85–1.03)	0.93	(0.84–1.03)	0.94	(0.86–1.05)	0.94	(0.85–1.04)
1/week	0.97	(0.85–1.10)	0.97	(0.85–1.10)	1.02	(0.89–1.16)	1.07	(0.94–1.22)
Couple of times/week	0.93	(0.83–1.05)	0.92	(0.81–1.04)	0.97	(0.85–1.10)	1.01	(0.89–1.15)
1/day or almost daily	0.91*	(0.83–1.00)	0.86***	(0.71–0.95)	0.91*	(0.82–1.00)	0.97	(0.87–1.07)
>1/day	0.90**	(0.82–0.99)	0.82***	(0.74–0.92)	0.87**	(0.78–0.98)	0.92	(0.82–1.03)

The N and Events (E) of Model 4 are $N=15,627$; $E=3679$ for prayer and $N=15,760$; $E=3716$ for participation

*, **, *** denote the significance level at 10%, 5% and 1%, respectively. Model 1 adjusts for age and gender. Model 2 adjusts for factors in Model 1 + education, household finances, depression, No. chronic conditions, BMI, ADL, and IADL. Model 3 adjusts for factors in Model 2 + country of residence. Model 4 adjusts for factors in Model 3 + potential mediators, namely, marital status, social support, smoking, physical inactivity, and alcohol consumption

## Discussion

### Main findings and existing literature

On this large sample of older European adults, reporting to follow a religion was associated with a decreased risk in mortality, and more so in males. This was true for Protestant, Catholic, and Orthodox Christians in some specifications. Muslims, on the other hand, may be at an increased risk, but results are inconclusive. Participation in a religious organisation exhibited a clear reduction in mortality risk and more so in males. A dose–response relationship was also observed, as was also the case with frequency of prayer, but less emphatically. Additionally, significant differences in survival between religious affiliations were detected, but a good portion of the them can be attributed to factors other than religion.

Since the participation in a religious organisation question is sort of the equivalent of church attendance but for every religion, this study replicates previous research findings that demonstrated a prophylactic association between church attendance and mortality in predominately (or exclusively) Christian samples (Ahrenfeldt et al. 2023; Bagiella et al. 2005; Bruce et al. 2022; Gillum et al. 2008; Hill et al. 2005; Hummer et al. 1999; Idler et al. 2017; Kim et al. 2015; La Cour et al. 2006; Li et al. 2016; Musick et al. 2004; O’Reilly and Rosato 2008; Schnall et al. 2010; Sullivan 2010; Teinonen et al. 2005; VanderWeele et al. 2017). The existence of a dose–response corroborates that the mechanisms affecting survival through religion respond to higher religious adherence.

Moreover, unlike Kim et al. (2015) and VanderWeele et al. (2017), this study finds a significant negative association between frequency of prayer and mortality for the highest frequencies. In addition, a modification effect by gender on survival was found but not for women, as was the case with religious participation in China (Zhang 2008), US (McCullough et al. 2009; Teinonen et al. 2005) and Denmark (Ahrenfeldt et al. 2023). In this study, males had lower risk than females both in religious following and religious participation exposures.

Regarding religious affiliations, this study accords with previous research from US (Idler et al. 2017; Kim et al. 2015) about a null effect in the Jewish population^5^, but it also adds to the inconclusiveness about Catholics. It does provide though some evidence for a reduced death rate in Protestants, not found previously. The antithetic results for specific religions when examining the median survival, unadjusted, and adjusted hazard ratios, is a sign that factors other than religion are responsible for the disparities in the survival of these populations. Minority religions within countries may suffer from exposure to discrimination, socioeconomic disadvantages, and acculturation stress, all of which can affect their survival (Aksoy et al. 2021). The observed Orthodox paradox, that is, Orthodox Christians having the longest median survival but HRs above one when controlling for country, is probably due to positivity violations (i.e. some countries lack specific religious followers). The sensitivity analysis shows that larger denominations are not affected by the inclusion of country fixed-effects.

### Mediation

Model 4 aimed at blocking the indirect paths by adjusting for potential mediators. Although this method in PH models cannot measure the size of mediation, it can perhaps provide a test for its existence (Wang and Albert 2017). Alcohol and tobacco consumption, as well as marital status, are dictated by some religions. While there is consensus that alcohol and tobacco consumption are detrimental to health and their mediating role is clear, for marital status things are more opaque. Some evidence exists that divorce is a risk factor for premature death, especially for men (Martin et al. 2005), and religion has been shown to increase marital satisfaction and commitment, as well as lower the rates of divorces (Mahoney et al. 1999).

While for religious affiliation and prayer frequency Model 4 attenuated the association towards the null and made it statistically insignificant, this was partly the case with religious participation. An attenuation towards the null did happen, but the negative association remained significant despite attempts to block potential paths. Of course that is no proof of a ‘direct effect’ since other potential mediators such as diet, fasting practices, and self-discipline^6^ (data not available), as well as measurement errors in the included variables may explain the remaining association.

### Strengths and limitations

A strength of this study is that it uses both frequency of prayer and religious participation frequency as a measure for religiousness. Previous approaches relied mostly on church, or service attendance, to capture the intensity of faith. A problem with this approach is that attendance to this events may have an obligatory character in some households, or be the product of habit, or just a social convention. Therefore, severe error may be present in the measurement of religiosity levels. Furthermore, studies have shown that the self-reported church attendance from surveys is significantly higher that the actual level (Hadaway et al. 1993). Prayer on the other hand, although not independent of church attendance, can take place in any location and is for the most part voluntary and more independent and private as an act. Unfortunately, in heterogeneous samples praying practices differ significantly and results are more difficult to interpret.

The main argument against this measure is that the frequency of prayer increases as health deteriorates^7^—assuming they pray for themselves and not for somebody else—and therefore, health status becomes a clear confounder in survival studies (McCaffrey et al. 2004). By controlling for physical and mental health, as was done in this study, this problem can be alleviated. Nevertheless, the presence of several religions with different praying locations and frequencies in the sample limits the interval validity, despite the fact that Christianity dominates the sample.

Although this sample is of one the largest used to study this subject in Europe, some religions such as Islam are underrepresented. This raises concerns about the sampling process and the response rates of these individuals despite the fact the this is a European sample—although Israel is not technically a European country—and the prevalence of Muslims is low. It cannot be determined from the data whether individuals who chose to participate from specific religions are representative followers of each religion, nor can be claimed that the sample is representative of older European adults. Moreover, some religions were completely missing from the data, and therefore, were not studied.

The small religion samples also undermine the statistical power and can cause severe positivity violations, especially when controlling for the country of residence (i.e. some countries lack specific religious followers). Consequently, since the country of residence is a clear confounder, one has to choose between unconfoundedness and reliance on model extrapolations, or greater positivity with confounding. Models 2 and 3 were designed to provide an estimate of this potential problem.

Another issue is that all data were self-reported; hence, an amount of measurement error is expected. The questions regarding religious participation refer to four weeks prior to the interview. This can make measurement sensitive to the time of the interview, although the latter is probably random. Since SHARE is not a cohort study with a proper follow-up but rather a longitudinal survey, missing values do not allow the use of time-varying exposures and force censoring (or elimination) when the time of death in not known. This might be a problem when studying religion since Argue et al. (1999) has provided evidence (from a US sample) that religion and religiousness might change over time, with people becoming more religious as they age. On the other hand, Coleman et al. (2004) provide some evidence of the opposite (i.e. they become less religious) from English adults; hence, the average effect of age on religiosity could be closer to the null. Nevertheless, given that the participants in our study were over the age of 50 when the religion information was recorded, it is perhaps safe to assume that denomination and religiousness did not change until their exit.

## Conclusions

This study points to the fact that active and frequent participation in a religious organisation is associated with increased survival in European older adults, and especially males. Simply following a religion or praying frequently may have less of a profound positive effect on longevity. From a public health perspective, given the amount and quality of evidence, religious participation should be encouraged for those that identify with a religion (VanderWeele et al. 2021).

Although differences in the median survival between followers of religions can be largely attributed to socioeconomic factors, especially in minority affiliations, the presence of health disparities between followers of different religions emerges as a topic for future research. Moreover, it will be interesting to see whether the ongoing secularisation of Europe and transition to godless societies will influence the health-religion association in younger cohorts. A new research challenge, that is a consequence of secularisation, is spiritual individuals who do not follow any religion, but have most of the mediating paths through which religion can influence health (e.g. diet, meditation, etc.) (Ransome 2020). Therefore, it would be probably wise if future researchers focused more on spirituality and less on the study of religion as a social determinant of health in Europe.

## Data Availability

The datasets used for this analysis are available from the SHARE website, http://www.share-project.org/home0.html.

## Footnotes

1 See Morton et al. (2017) for a thorough discussion on the pathways between religion and health.

2 The limitations concern the measurement of religiosity levels. For example, Islam has a much higher daily prayer frequency than Christianity. This would automatically classify a Muslim participant as more religious when that might not be the case. This is not a major issue in our study since Muslims are only a tiny fraction of the sample and most religions (and as % of the sample) have more or less comparable prayer frequencies. The limitations of church attendance are discussed later in the text.

3 More details on the survival data extraction methods can be found in Christopoulos et al. (2022).

4 These differences are not expected to be of a magnitude that would make adjustments insufficient to achieve conditional exchangeability, or result in artificially unequal deaths between the compared groups.

5 A study of Israeli males does show a protective association of religiosity against all-cause mortality (Eilat-Adar et al. 2022)

6 There is the opinion than religiousness influences healthy behaviours by providing individuals with self-control and discipline (McCullough and Willoughby 2009) and some even claim that there is even a bidirectional association, that is, health behaviour also steers people towards religion (McCullough et al. 2009).

7 A negative association between prayer frequency and health outcomes was indeed found by Hank and Schaan (2008) who examined data from the same survey.
